# Distinct Impacts of *Eda* and *Edar* Loss of Function on the Mouse Dentition

**DOI:** 10.1371/journal.pone.0004985

**Published:** 2009-04-02

**Authors:** Cyril Charles, Sophie Pantalacci, Paul Tafforeau, Denis Headon, Vincent Laudet, Laurent Viriot

**Affiliations:** 1 Institut International de Paléoprimatologie Paléontologie Humaine: Evolution et Paléoenvironnements UMR CNRS 6046, Université de Poitiers, Poitiers, France; 2 Team 〈〈 Molecular Zoology 〉〉, Institut de Génomique Fonctionnelle de Lyon, Université de Lyon, UMR CNRS 5242, INRA, Université Claude Bernard Lyon 1, Ecole Normale Supérieure de Lyon, Lyon, France; 3 European Synchrotron Radiation Facility, Grenoble, France; 4 The Roslin Institute and Royal (Dick) School of Veterinary Studies, University of Edinburgh, Edinburgh, United Kingdom; 5 Team 〈〈 Evo-Devo of Vertebrate Dentition 〉〉, Institut de Génomique Fonctionnelle de Lyon, Université de Lyon, UMR CNRS 5242, INRA, Université Claude Bernard Lyon 1, Ecole Normale Supérieure de Lyon, Lyon, France; Max Planck Institute for Evolutionary Anthropology, Germany

## Abstract

**Background:**

The Eda-A1-Edar signaling pathway is involved in the development of organs with an ectodermal origin, including teeth. In mouse, mutants are known for both the ligand, Eda-A1 (Tabby), and the receptor, Edar (Downless). The adult dentitions of these two mutants have classically been considered to be similar. However, previous studies mentioned differences in embryonic dental development between *Eda* ^Ta^ and *Edar* ^dl-J^ mutants. A detailed study of tooth morphology in mutants bearing losses of functions of these two genes thus appears necessary to test the pattern variability induced by the developmental modifications.

**Methodology/Principal Findings:**

3D-reconstructions of the cheek teeth have been performed at the ESRF (Grenoble, France) by X-ray synchrotron microtomography to assess dental morphology. The morphological variability observed in *Eda* ^Ta^ and *Edar* ^dl-J^ mutants have then been compared in detail. Despite patchy similarities, our detailed work on cheek teeth in *Eda* ^Ta^ and *Edar* ^dl-J^ mice show that all dental morphotypes defined in *Edar* ^dl-J^ mice resolutely differ from those of *Eda* ^Ta^ mice. This study reveals that losses of function of *Eda* and *Edar* have distinct impacts on the tooth size and morphology, contrary to what has previously been thought.

**Conclusion/Signifiance:**

The results indicate that unknown mechanisms of the Eda pathway are implicated in tooth morphogenesis. Three hypotheses could explain our results; an unexpected role of the Xedar pathway (which is influenced by the *Eda* gene product but not that of *Edar*), a more complex connection than has been appreciated between Edar and another protein, or a ligand-independent activity for Edar. Further work is necessary to test these hypotheses and improve our understanding of the mechanisms of development.

## Introduction

Genes of the Eda-A1/Edar signaling pathway are involved in the development of organs with an ectodermal origin, such as hair, glands, teeth [1]–[3] and palatal rugae [4]. The *Eda* gene, carried by the X chromosome, encodes the ligand (Eda-A1), and the *Edar* gene encodes its receptor (Edar). *Eda* ^Ta^ (Tabby) and *Edar* ^dl-J^ (Downless) mutant mice bear loss-of-function mutations for the *Eda* (ectodysplasinA) and the *Edar* (ectodysplasinA-receptor) genes, respectively [5], [6]. Consistent with their operation in a linear signalling pathway, the ectodermal organs of these mutants display similar gross phenotypes. However, differences between *Eda* and *Edar* mutants in the histological structure of the submandibular salivary gland have been reported [7]. Concerning dental morphology, only the dentition of *Eda* mutants has been deeply investigated. These studies revealed a high morphological diversity of the cheek dentition, characterized by modifications in the number of teeth and in the number and arrangement of cusps for homozygous and heterozygous mice [1], [8]–[13]. In contrast, no study has described the *Edar* ^dl-J^ dental phenotype, which is usually supposed to display the same dental defects as in *Eda* ^Ta^ mice [14]. However, differences between these mutants have been detected in the enamel knots, which are transient signalling centres that define the cusp pattern of the mature tooth [15]. The enamel knots of *Eda* ^Ta^ embryonic teeth are simply smaller in than those of WT mice [16], while *Edar* ^dl-J^ mutant molars have a structure termed the ‘enamel rope’ which is composed of enamel knot cells that are extended across the tooth primordium due to a failure of cell condensation [17]. This may indicate that *Eda* and *Edar* losses of function are likely to have different consequences on mature dental morphology. This prompted us to study the cheek dentition in *Edar* ^dl-J^ mutant mice to determine the extent to which *Eda* ^Ta^ and *Edar* ^dl-J^ phenotypes are similar and to discuss the implications of their putative differences and similarities.

## Materials and Methods

### Downless^j^ and Tabby mice

The *Edar* ^dl-J^ mice (FVB background) have been bred at the PBES of IFR 128 (Lyon). These mice carry a G to A transition mutation causing a glutamate to lysine substitution in the death domain of the Edar protein (E379K) [6]. Old studies on *Edar* mutant mice used either *Edar* ^dl^ [1], [9] or *Edar* ^sleek^ [3] mice. *Edar* ^dl-J^ mice have been used in more recent studies comparing the dental development and functional morphology of *Eda* and *Edar* mutant mice [17]. Comparison of *Edar* ^dl-J^ and *Edar* ^sleek^ dental morphology showed no differences [17]. Homozygous *Eda* and *Edar* mutant mice were identified according to external morphological criteria, such as the bald spot behind ears. Heterozygous *Eda* (female) mice were identified morphologically by the distinctive striping of the coat that gave these mutants their original name, ‘Tabby’. Heterozygous and wild-type *Edar* ^dl-J^ specimens exhibit similar external traits and were genotyped through PCR amplification of a 306 bp fragment covering the point mutation (primers: 5′ GTCTCAGCCCCACCGAGTTG and 3′ GTGGGGAGGCAGGTGGTACA), followed by sequencing. The *Edar* ^dl-J^ sample was composed of 20 heterozygous (*Edar* ^dl-J/+^), 47 homozygous (*Edar* ^dl-J^) and 5 control (WT) specimens. The Tabby sample used in comparison overlaps the sample studied by Kristenova et al. [10]. They carry the *Eda* ^Ta^ null allele of the *Eda* gene, carried by the X-chromosome. *Eda* ^Ta^ mice are on a mixed background (C57Bl6J+CBA), the sample included 60 heterozygous females (*Eda* ^Ta/+^), 23 homozygous (*Eda* ^Ta/Ta^) or hemizygous females (*Eda* ^Ta/0^, which have a single X chromosome and display the same phenotype as homozygous [10]), 57 hemizygous males (*Eda* ^Ta/Y^) and 40 WT mice. Mice were killed by cervical dislocation. The experimental protocol was designed in compliance with recommendations of the EEC (86/609/CEE) for the care and use of laboratory animals.

The uncertain homology of teeth between WT and mutant mice led us to adopt a nomenclature using T^x^ and T_x_ where T and x respectively symbolize the tooth and its rank within the row (e.g. T^1^ for the first upper cheek tooth, T_1_ for the first lower cheek tooth).

### 3D-data acquisition

Cheek teeth were examined using a Leica MZ16 stereomicroscope. Morphotypes have been defined for lower and upper tooth rows on the basis of the number and arrangement of cusps. Occlusal surface areas of cheek teeth were measured from digitized pictures using Optimas software. It has been demonstrated that X-ray synchrotron microtomography brings very high quality results for accurate imaging of small teeth [18]. Thus, tooth rows of a representative panel which covers the totality of observed morphologies were imaged using X-ray-synchrotron microtomography at the European Synchrotron Radiation Facility (ESRF, Grenoble, France). 3D-renderings were performed using VGStudiomax software.

### Statistical tests

Statistical tests have been performed to compare tooth size. The Kruskal-Wallis analysis of variance was used to verify the significance of observed differences between *Eda* ^Ta^ and *Edar* ^dl-J^ mice. This is a non-parametric method, which tests equality of population medians among groups. The non-parametric Wilcoxon rank-test is used to indicate which groups are statistically different from the others.

## Results

### Wild-type mice

WT specimens from *Eda* ^Ta^ and *Edar* ^dl-J^ breeding colonies all display the normal dental morphology, without anomalies, and measurements show similar tooth size (ANOVA test at p = 0.05 threshold value). Thus, molars from WT from both genetic backgrounds were included in a single WT sample.

### Heterozygous *Eda* ^Ta/+^ and *Edar* ^dl-J/+^ mice

**Figure 1:**
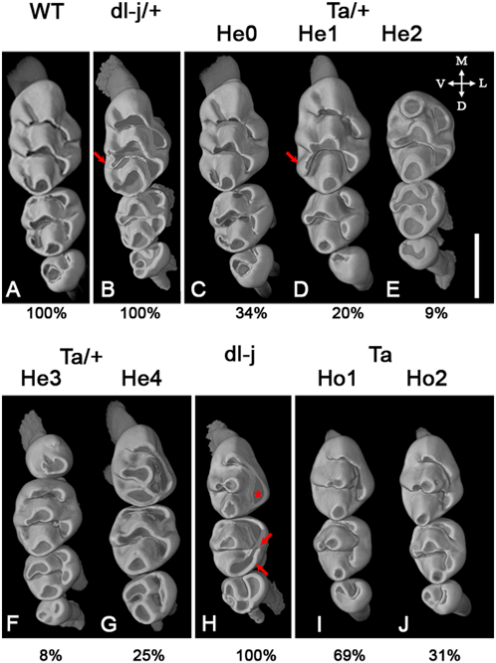
Upper tooth rows; wild-type morphology and morphotypes defined among *Eda^Ta^* and *Edar^dl-J^* mutant mice. A: WT morphology, B: *Edar^dl-j/+^* morphology; C: morphotype Ta He0; D: morphotype Ta He1; E: morphotype Ta He2; F: morphotype Ta He3; G: morphotype Ta He4; H: *Edar^dl-j^* morphology; I: morphotype *Eda^Ta^* Ho1; J: morphotype *Eda^Ta^* Ho2. The proportions indicated below the morphotypes are the occurrence frequency of the morphotypes. Images are obtained using X-ray synchrotron microtomography. Tooth orientation: M: mesial, D: distal, V: vestibular, and L: lingual. Scale bar: 1 mm. The structures indicated by arrows and asterisk are discussed in the main text.

**Figure 2:**
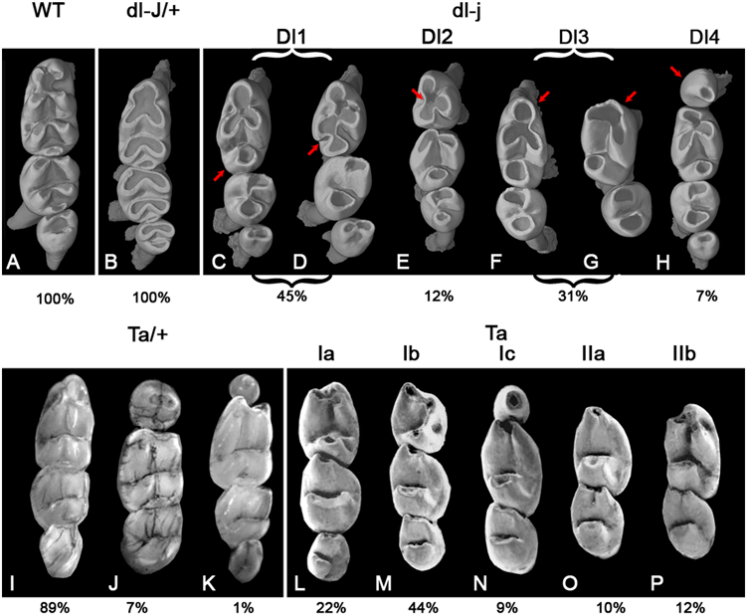
Lower tooth rows; wild-type morphology and morphotypes defined among *Eda^Ta^* and *Edar^dl-J^* mutant mice. A: WT morphology; B: *Edar^dl-J/+^* morphology; C–D: *Edar^dl-j^* morphotype Dl1, E: *Edar^dl-j^* morphotype Dl2, F–G: *Edar^dl-j^* morphotype Dl3, H: *Edar^dl-j^* morphotype Dl4, I–K: *Eda^Ta^* morphotypes, L: *Eda^Ta^* morphotype Ia, M: *Eda^Ta^* morphotype Ib, N: *Eda^Ta^* morphotype Ic, O: *Eda^Ta^* morphotype IIa, P: *Eda^Ta^* morphotype IIb. The proportions indicated below the morphotypes are the occurrence frequency of the morphotypes. Images of *Edar* ^dl-J^ mice are obtained using X-ray synchrotron microtomography. Images of *Eda* ^Ta^ mice are obtained by photography and are taken from Kristenova et al. [10] and Peterkova et al. [30]. Same orientation as in Fig. 1. The structures indicated by arrows are discussed in the main text.

**Figure 3:**
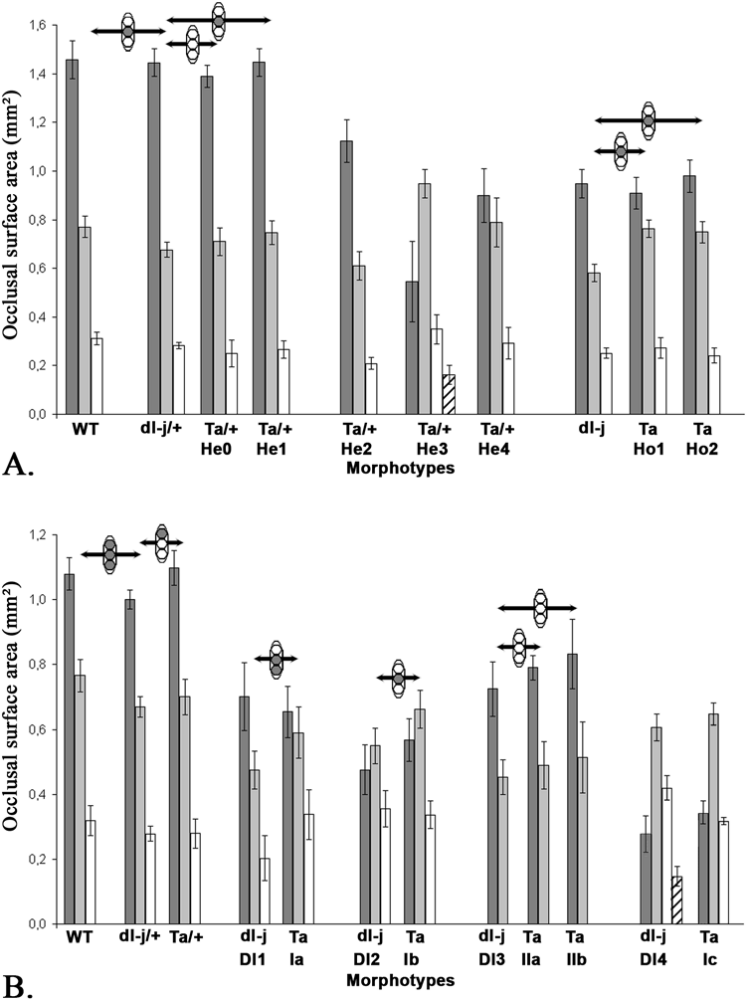
Size of the cheek teeth of Wild-type, Downless and Tabby heterozygous (+/−) and homozygous (−/−) mice. A. Upper cheek teeth. Dark grey: T^1^, light grey: T^2^, white: T^3^, and hatched: T^4^. B. Lower cheek teeth. Dark grey: T_1_, light grey: T_2_, white: T_3_, and hatched: T_4_. Black bars indicate the standard-deviation of the mean. WT: Wild-type mice, dl-J/+: *Edar^dl-J/+^*, Ta/+: *Eda^Ta/+^*, dl-J: *Edar^dl-J^*, Ta: *Eda^Ta^*. The arrows indicate the statistical size comparison between morphologically close morphotypes, the three circles on each arrow represent from top to bottom the first, second and third cheek teeth, a grey-filled circle indicates a statistically significant difference in size between the two considered teeth according to a Wilcoxon rank-test at p = 0.05 threshold value, a white circle indicates the absence of statistical difference. Morphotypes that appear to be obviously different on the plot were found to have statistically significant differences.

As *Eda* is located on the X chromosome while *Edar* is on an autosome, morphological differences linked to the X-inactivation process were predictable. Accordingly, in both the upper and lower cheek dentitions, heterozygous *Edar* ^dl-J/+^ mice display dental morphologies very close to WT specimens (Fig. 1A–B, Fig. 2A–B) whereas *Eda* ^Ta/+^ mice dentitions exhibit highly variable patterns (Fig. 1C–G, Fig. 2I–K). *Edar* ^dl-J/+^ upper tooth rows differ from the WT morphology by the occurrence of a supplementary centro-vestibular cusp at the T^1^ (arrow on Fig. 1B). This pattern approaches the morphotype He1 of *Eda* ^Ta/+^ mice (arrow on Fig. 1D) [13]. The size of the upper teeth only differs between WT, *Eda* ^Ta^ He1 and *Edar* ^dl-J^ by a smaller T^2^ in *Edar* ^dl-J^ (Fig. 3A). Upper tooth size is similar for *Edar* ^dl-J/+^ and *Eda* ^Ta/+^ He0, defined as the Tabby heterozygous morphotype exhibiting the wild-type morphology (presented Fig. 1C).

*Eda* ^Ta/+^ mice sometimes exhibit a small mesial lower tooth (Fig. 2J–K) which is never found in *Edar* ^dl-J/+^ mice. Moreover, *Edar* ^dl-J/+^ lower teeth are all smaller than those of the WT. They also differ from the *Eda* ^Ta/+^ major morphotype by a smaller T_1_ (Fig. 3B). The almost normal morphology of upper and lower cheek dentitions of *Edar* ^dl-J/+^ is thus far from the morphological diversity observed in *Eda* ^Ta/+^.

### Homozygous *Eda* ^Ta^ and *Edar* ^dl-J^ mice

Upper and lower cheek teeth clearly differ between *Eda* ^Ta^ and *Edar* ^dl-J^ mice in terms of morphology and size. *Edar* ^dl-J^ upper dentition is characterised by: **(i)** a T^1^ with a single lingual cusp (asterisk in Fig. 1H) linked by a crest to the mesial-most cusp, **(ii)** a T^2^ with a lingual interconnection between mesial and distal cusps (arrows in Fig 1H). T^1^ of *Edar* ^dl-J^ are similar to those of *Eda* ^Ta^ Ho1 (Fig. 1H–I). T^1^ of *Edar* ^dl-J^ and *Eda* ^Ta^ Ho1 statistically have the same size (Fig. 3A). However, T^2^ are statistically smaller in *Edar* ^dl-J^ (Fig. 3A) and the lingual ridge of *Edar* ^dl-J^ T^2^ does not occur in *Eda* ^Ta^ T^2^. The morphological differences between *Eda* ^Ta^ and *Edar* ^dl-J^ T^2^ can be explained by their crown size differences referring to the ‘patterning cascade mode of cusp development’ [19] in which the signaling centre succession, and consequently the number and position of cusps, is linked to the crown size.

The lower cheek dentition of *Edar* ^dl-J^ is more variable than the upper. Four morphotypes can be defined (Fig. 2C–G), specimens can exhibit two different morphotypes on left and right sides, none of the four morphotypes occurs in *Eda^Ta^* dentition. **(i)** The first morphotype, Dl1 (for Downless1), includes 45% of the studied material. It is characterized by a three-toothed row and a four-cusp T^1^. The three mesial cusps form a three-leaf clover shape, while the fourth cusp is either isolated in the distal part of the tooth (85% of Dl1 tooth rows, arrowed in Fig. 2C) or connected to the others by a longitudinal crest (15% of Dl1 tooth rows, arrowed in Fig. 2D). No *Eda* ^Ta^ morphotype is similar to this Dl1 morphotype. **(ii)** The Dl2 morphotype (12% of examined tooth rows) also exhibits three cheek teeth, but the T_1_ only displays three cusps. As in Dl1 morphotype, the cusps form a three-leaf clover shape connected in the centre of the occlusal surface (arrow in Fig. 2E) but the distal cusp is missing. Except for cusp connections, the Dl2 morphotype approaches the *Eda^Ta^* Ib morphotype (Fig. 2M), but differs by the size of the T_2_ (Fig. 3B). **(iii)** The Dl3 morphotype (31% of tooth rows) is characterized by rows with two lower cheek teeth (Fig. 2F–G). The T_1_ of the Dl3 morphotype resembles this of the Dl1 morphotype (Fig. 2C,F). However, the three mesial cusps display highly variable size and position. The most mesial element varies from a large rounded cusp (arrowed in Fig. 2F) to a highly reduced, almost absent, element (arrow in Fig. 2G). This latter morphology is alike the *Eda* ^Ta^ IIb morphotype (Fig. 2P). The Dl3 morphotype displays the same tooth size as *Eda* ^Ta^ IIa and IIb morphotypes (Fig. 3B). **(iv)** The Dl4 morphotype (7% of tooth rows) is characterized by a small T_1_ (arrow in Fig. 2H), 57% of the Dl4 tooth rows encompass three teeth, while 43% have a tiny T_4_. The T_2_ encompasses four cusps, three mesial interconnected and an isolated distal one. The presence of the reduced T_1_ is reminiscent of the *Eda* ^Ta^ Ic morphotype (Fig. 2N). However, the morphology of the T_1_ is highly different as it encompasses a higher number of cusps than that of the *Eda* ^Ta^ Ic morphotype, which moreover never exhibits 4 teeth.

## Discussion

### X-inactivation might explain differences between heterozygous *Eda* ^Ta/+^ and *Edar* ^dl-J/+^ mice

The many observed differences, and the higher variability of tooth rows, between *Eda* ^Ta/+^ and *Edar* ^dl-J/+^ mice can be explained by the fact that the *Eda* gene is X-linked and that *Edar* is carried by an autosome. Due to the X-inactivation effect in females [20], [21], *Eda^Ta/+^* mice are mosaics of cells with expression of a wild-type or a null *Eda* gene. This might induce a strong and random variability in the amount of Ectodysplasin-A1 protein available for dental development, explaining the higher morphological variability recorded in *Eda* ^Ta/+^. This phenomenon does not occur in the *Edar* ^dl-J^ teeth since the gene is located on an autosome (chromosome 10).

### Differences between homozygous *Eda* ^Ta^ and *Edar* ^dl-J^ mice imply other mechanisms

The same general trends in the reduction of the cusp number and tooth size are observed in both *Eda* ^Ta^ and *Edar* ^dl-J^ mutants. However, none of the *Eda* ^Ta^ and *Eda* ^dl-J^ tooth rows are identical. The two mutants are supposed to display total losses of function of the ligand and the receptor of the Eda pathway, respectively. According to Tucker et al. [17], *Eda* mice have small enamel knots while *Edar* ^dl-J^ mice have disorganised “enamel ropes”. As enamel knots are orchestrating the final mineralized crown pattern, differences of size and morphology, differences in enamel knots are likely to explain differences of size and morphology between *Eda* ^Ta^ and *Edar* ^dl-J^ dentitions.

Various hypotheses could explain these differences: **(i)** They could be linked to epistatic differences between the two different backgrounds of *Eda* ^Ta^ and *Edar* ^dl-J^ mice (respectively mixed C57Bl6J+CBA and FVB). However we consider this possibility to be unlikely to be the only explanation. Indeed, suppression of Eda action in embryonic CD-1 mice by addition of soluble forms of Edar to tooth explants results in the same phenotype as seen in *Eda* ^Ta^ mice on NMRI and mixed CBAT6T6xNMRI backgrounds but differs from the *Edar* ^dl-J^ on CD-1 background [17]. This indicates that differences observed between enamel knots in *Eda* ^Ta^ and *Edar* ^dl-J^ mice are not due to a difference in background but rather to an intrinsic difference between loss of *Eda* function and alteration of *Edar* function in *Edar* ^dl-J^. **(ii)** *Eda* codes for two proteins, Eda-A1, which binds to Edar, and Eda-A2, which binds to Xedar [22]. The Xedar pathway is thus lost in *Eda* ^Ta^ mice but still present in *Edar* ^dl-J^ mice. An effect on tooth development of the loss of the Xedar pathway in the case of *Eda* loss of function and not in the *Edar* one might explain some of the differences that we report. Though a study of Xedar-null mice indicated no requirement for this gene in the normal development of ectodermal organs [23], dominant negative and constitutively active forms of this protein have been shown to have effects similar to those of Edar in developing chicken skin [24] and a compensatory action of Xedar that is revealed only upon loss of Edar function can not be ruled out. **(iii)** The *Edar* ^dl-J^ allele may not be a null mutation. Indeed, the *Edar* ^dl-J^ mutation causes a substitution in the death domain of the Edar protein, which connects to the Edar adapter with death domain, Edaradd [25]. However the intracellular domain also contains two sites for direct binding of TRAF proteins that are conserved among vertebrates [26]. These sites may allow Edaradd-independent signaling, even if, to date, no molecular study indicates utilisation of such an alternative pathway by Edar. Alternatively, the *Edar* ^dl-J^ mutants EdarE379K protein may retain some interaction with Edaradd allowing transmission of a weak signal through this pathway. Assays in cultured cells have given conflicting results on the nature of the signal output of EdarE379K. Yan et al. [22] reported that EdarE379K fails to activate NF-B, while Kumar et al. [27] found that the mutant protein retains a significant capacity to stimulate this pathway. Thus this hypothesis remains an attractive possibility that has to be experimentally tested. A complete loss of function of the gene may, in that case, reveal morphological differences with the present Downless genotype used in this study **(iv)** A ligand-independent activation mechanism for *Edar* could also explain the morphological differences observed. Indeed, the finding that elevation of *Edar* expression can rescue the hair phenotype of *Eda* ^Ta^ mutant embryos [28] suggests that Edar does undergo ligand-independent signaling, at least when expressed at sufficiently high levels. Alternatively, some transmembrane receptors are dependence receptors, such as the Netrin-1 receptor which displays ligand-independent activities [29]. Under this hypothesis, the absence of Eda that leads to a pathway containing an “active” unliganded Edar will lead to a different phenotype than the loss of *Edar*. This hypothesis could be tested by crossing *Edar* ^dl-J^ with *Eda* ^Ta^ mice. Such a cross should result in an *Edar* ^dl-J^ type phenotype. Another test could be a cross of Tabby mice with a loss-of-function mutant of the *Edaradd* gene that should also give rise to an *Edar* loss-of-function phenotype.

To sum up, loss of function of *Eda* or *Edar* leads to different dental morphologies, presumably linked to the differences in their enamel knot morphologies. These differences indicate unknown mechanisms of the Eda pathway involved in tooth morphogenesis. These unknown mechanisms we hypothesize to be based on an unexpected role of the Xedar pathway in tooth development, a more complex connection than thought between Edar and Edaradd, or on a ligand-independent activity for Edar.
